# A Rapid Literature Review of Multi-Criteria Decision Support Methods in the Context of One Health for All-Hazards Threat Prioritization

**DOI:** 10.3389/fpubh.2022.861594

**Published:** 2022-04-15

**Authors:** Jiawei Zhao, Tiffany Smith, Melissa Lavigne, Cécile Aenishaenslin, Ruth Cox, Aamir Fazil, Ana Johnson, Javier Sanchez, Benoit Hermant

**Affiliations:** ^1^Risk and Capability Assessment Unit, Public Health Agency of Canada, Ottawa, ON, Canada; ^2^Department of Pathology and Microbiology, University of Montreal, Montreal, QC, Canada; ^3^Centre de recherche en santé publique de L'Université de Montréal et du CIUSSS du Centre-Sud-de-L'Île-de-Montréal, Montréal, QC, Canada; ^4^Department of Health Management, Atlantic Veterinary College, University of Prince Edward Island, Charlottetown, PE, Canada; ^5^National Wildlife Management Centre, Animal and Plant Health Agency, Woodchester Park, United Kingdom; ^6^National Microbiology Laboratory, Public Health Agency of Canada, Ottawa, ON, Canada; ^7^Department of Public Health Sciences, Queen's University, Kingston, ON, Canada

**Keywords:** MCDA, rapid review, all-hazards, One Health, prioritization, public health, multi-criteria decision analysis, decision support

## Abstract

**Background:**

Multi-Criteria Decision Analysis (MCDA) is a decision support tool that can be used in public health emergency management. The use of a One Health lens in MCDA can support the prioritization of threats and interventions which cut across the human, animal, and environmental domains. Previous literature reviews have focused on creating a snapshot of MCDA methodological trends. Our study provides an update to the MCDA methods literature with key considerations from a One Health perspective and addresses the application of MCDA in an all-hazards decision-making context.

**Methods:**

We conducted a literature search on MEDLINE, EMBASE, SCOPUS, the CAB database, and a limited online gray literature search in partnership with a librarian from Health Canada. Articles were limited to those published in the year 2010 or later in a high-income setting (OECD member countries).

**Results:**

Sixty-two articles were included for synthesis. Of these articles, most were Canadian studies (20%); and prioritized health risks, threats, and interventions in the human domain (69%). Six commonly used prioritization criteria were identified: threat, health, intervention, strategic, social, and economic impact. Stakeholders were engaged in 85% of studies and commonly consisted of government groups, non-governmental groups, subject matter experts, and the public. While most articles (65%) included elements of One Health based on our definition, only 5 studies (9%) explicitly acknowledged One Health as a guiding principle for the study. Forty seven percentage of studies noted that MCDA was beneficial in supporting the decision-making process.

**Conclusion:**

Current literature on health prioritization presents some variability in the depth of integration of the One Health framework and on the use of various MCDA methodologies given prioritization objectives. Studies which applied a comprehensive One Health approach, prioritized disparate threats, or conducted cyclical prioritizations for governing bodies were broad in scope, but sparse. The results of our review indicate the need for better guidance on the integration of a One Health approach and the use of various MCDA methods given the main prioritization objectives.

## Introduction

A key component of strategic emergency management is comparative risk assessment and threat prioritization (1). Threat prioritization methods within an all-hazards context must consider the full range of potential disasters, threats, or hazards for which preparedness and emergency response capabilities are required. Specific to public health, an all-hazards context examines a wide range of health threats and risks, including those increasing in frequency and complexity due to globalization, climate change, and terrorism. Moreover, the all-hazards approach to threat prioritization includes the capacity to evaluate disparate threats (e.g., influenza, flooding, bioterrorism etc.) and the examination of the interplay between multiple threats and their drivers. Thus, threat prioritization for strategic public health emergency management must enable multi-disciplinary collaboration, consideration of diverse evidence, and adaptability to shifts in decision-making requirements.

Paradigms such as One Health can provide a suitable approach to examine diverse public health threats and their drivers. The One Health paradigm recognizes the interconnectedness of human, animal, and environmental domains and thus the necessity to reflect on all three to prevent and control public health threats (2, 3). For example, emerging zoonotic diseases due to climate change can be analyzed through individual compartments like human, animal, and environmental health, or the effects that intersections of each component have on one another. Although valuable to the emergency management planning, coordination and policy development process, the integration of all-hazards risk assessment methods with a One Health approach adds a layer of complexity to an already challenging decision-making context.

Complex decision-making can be facilitated by decision-analysis: a systematic process that evaluates all aspects of a decision (4). Many methodological approaches are available in the field of decision-analysis varying in their complexity, transparency, and time requirement (5). MCDA provides a means of structuring complex decision-making processes conducted with multiple stakeholders and may be a suitable method for prioritizing all-hazard threats using a One Health approach. MCDA incorporates conflicting criteria which are critical characteristics of public health threats and One Health decision problems (3). MCDA methods also enable the development of decision-making frameworks that can be re-used and updated based on shifts in the decision-making context (6).

Previous literature reviews on the use of MCDA in health have focused on creating a snapshot of the trends and methodologies used for MCDA models (7, 8). Key findings from this past literature include an increasing number of published MCDA articles by year, an even distribution of weighting methods like Analytical Hierarchy Process (AHP), Multi-Attribute Utility Theory (MAUT), Technique for Order of Preference by Similarity to Ideal Solution (TOPSIS), and outranking being used, and Canada being the country with the most published MCDA articles (7, 8). In their paper, Frazão et al. highlighted a need for subsequent literature reviews to investigate the methodological applications of MCDA (8). Our study fulfills this gap in the literature and goes one step beyond addressing the methodological applications of MCDA in an all-hazards decision-making context by incorporating key considerations from a One Health perspective.

In this paper, we presented a rapid literature review examining how MCDA support methods are used for the prioritization of health-related risks, threats, and interventions in human, animal, and environmental decision problems. A rapid literature review was chosen in place of more resource-intensive literature review methods to synthesize key information required to support ongoing projects at the Public Health Agency of Canada in a timely manner. Our main objective was to identify key considerations and practices for the application of a One Health based MCDA approach for all-hazard threat prioritization to inform decision-making on public health emergency management in Canada. A particular focus was placed on identifying considerations related to the use of a One Health approach, the prioritization of disparate threats, and cyclical prioritizations to inform governing bodies. To structure our literature review, we followed the general framework for conducting rapid literature reviews as outlined by the 2020 Cochrane Rapid Reviews guidance document with slight modifications (9).

## Methods

### Article Identification

We conducted a literature search on the MEDLINE, EMBASE, SCOPUS, and CAB databases. The search strategy was developed in partnership with a librarian from the Public Health Agency of Canada and focused on three themes: decision support methods focusing on MCDA, studies conducting prioritization exercises, and health related studies in the human, animal, and environmental fields. The search strategy was refined iteratively in consultation with authors resulting in a second search strategy to capture papers missed in the first search round (Supplementary Material).

Specific research questions guiding our search include:

- What are the current decision-making support tools being used to guide public health decision-making?
- What types of alternatives are analyzed through MCDA (e.g., interventions, risks)?
- What methods are used in MCDA and other decision-making support tools to assess and weigh competing criteria?
- What stakeholder groups are commonly involved and how are stakeholders engaged throughout the MCDA process?
- To what extent are One Health principles integrated into MCDA models?

We limited our search to articles published in the context of an Organization for Economic Co-operation and Development (OECD) member country and written in the English or French language. Articles which conducted a literature review on MCDAs were excluded. Additionally, a date restriction was set for records published after January 1, 2010, a year in which multiple countries and international organizations recommended the adoption and broad implementation of One Health approaches (2). A subsequent limited online gray literature search was conducted by the same PHAC librarian who identified and recommended potential sources of gray literature on Google Scholar, the European Center for Disease Prevention website, the Agency for Healthcare Research and Quality website, and various other governmental websites. An independent reviewer retrieved relevant gray literature from these sources. Lastly, additional relevant literature was obtained through the Integrated Threat Assessment Methods (ITAM), a government-academia collaborative Think Tank whose members primarily comprise of professors from Canadian universities.

### Article Screening

All 1,098 records identified from database searching and other sources were first gathered on RefWorks online and then exported to Zotero reference manager. Records identified as conference papers, posters, books, or book sections (*n* = 49) were removed, and the remaining 1,049 records were then exported to Microsoft Excel *via* a.csv file for title and abstract screening.

**Figure 1:**
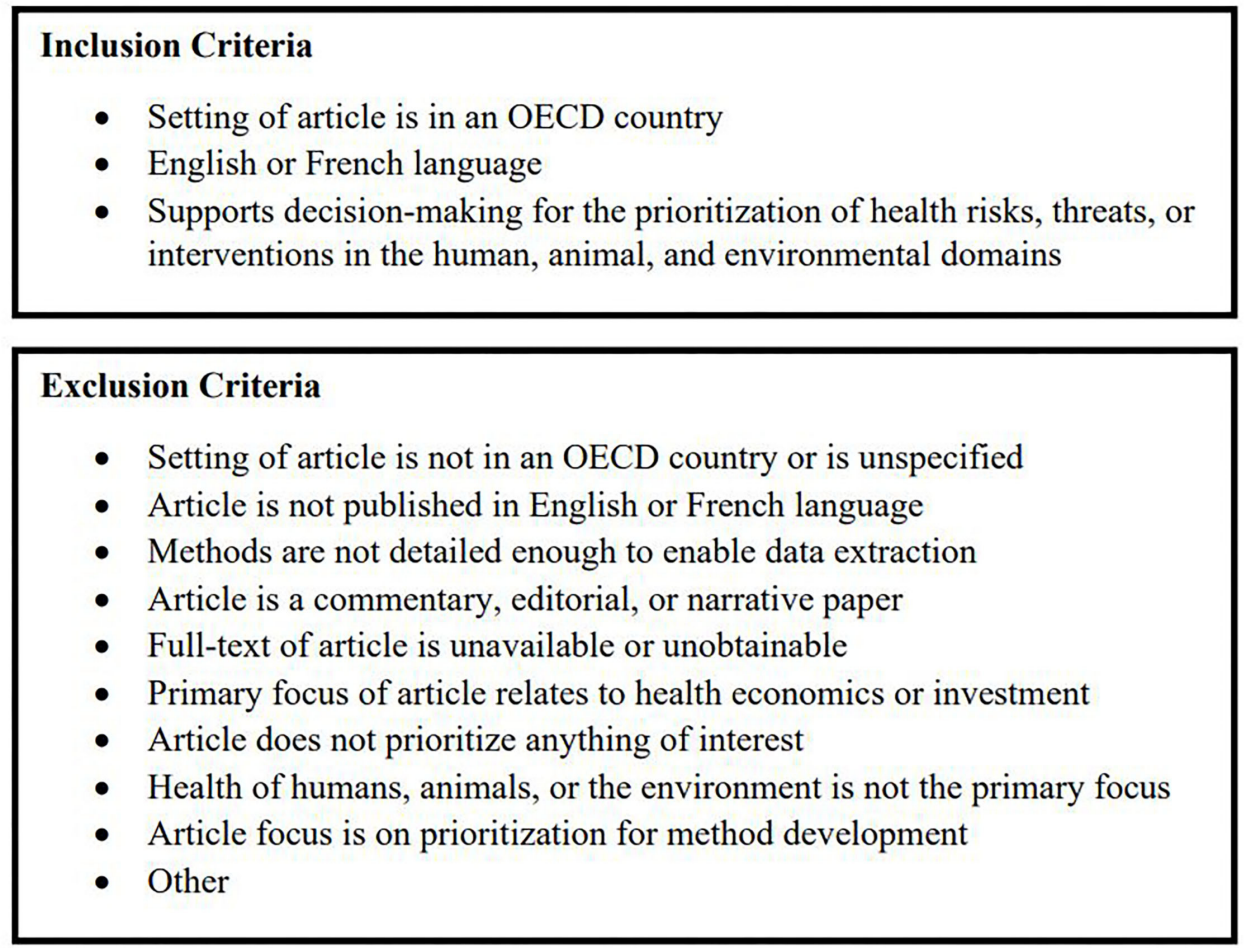
Inclusion and exclusion criteria.

To be included for review, an article had to describe the use of a decision support tool or methodology to prioritize a health-related initiative, the setting of the study had to be within an OECD member country, and the article had to be published in English or French language. Articles were excluded if they did not fit the inclusion criteria or if they met the predefined exclusion criteria: Articles that are not health focused (i.e., article focus was on industry, urban development, economics), articles that did not prioritize health threats or interventions, and articles with a use case specific to one population group (Figure 1). Two independent reviewers TS and JZ completed article screening.

Following the Cochrane Rapid Reviews protocol, 25 training articles were used for title and abstract screening and 5 training articles were used for full-text screening. Once sufficient confidence between the two reviewers was established, following a review of article screening and inclusion/exclusion criteria, the two reviewers proceeded independently for data extraction and synthesis.

### Data Extraction

The data extraction fields were as follows; study context (e.g., study objectives), study duration, analysis methods (e.g., type of decision support tool), criteria used to support decision making, approach to stakeholder engagement, and key findings in terms of prioritization methods. Study duration was defined based on any time or period referenced within the study, while also taking into consideration the date the article was submitted for publication.

Articles for data extraction were split between two independent reviewers JZ & TS. Studies using a One Health approach, prioritizing disparate threats, or involving cyclical prioritization for a governing body were flagged for additional analysis. We defined studies using a One Health approach as those in which focus, stakeholders, or criteria spanned across multiple domains. Studies with a One Health focus were those in which the threat, intervention, or the population at risk were in different domains (e.g., ranking municipal populations by vulnerability to heat waves). Disparate threats were defined as threats that were different in kind and not typically comparable in keeping with an all-hazards perspective on threat prioritization. Cyclical prioritization for governing bodies included those studies that presented a reusable method of prioritization for a decision-making authority.

Data extraction was completed independently by two reviewers TS and JZ using a pre-built form on Microsoft Excel. Each reviewer completed data extraction for half of the total pool of 54 non-companion articles. Following data extraction, reviewers independently completed a data quality check for all articles and consensus was reached through discussion.

### Synthesis of Studies

We conducted quantitative data analysis on Microsoft Excel using built-in formulas, Analysis ToolPak, and pivot tables to synthesize our findings. Quantitative analysis focused on summarizing study characteristics, method characteristics, and the number of articles by relevant descriptive categories. Qualitative findings were analyzed through thematic analysis. Thematic analysis on the reported limitations and benefits of using multi-criteria decision support methods was based on qualitative data extracted from the discussion sections of all papers (including companion papers).

## Results

In total, 1,239 articles were identified from database searching and 76 from gray literature searches. An additional 11 articles were recommended through consultation with members of ITAM. After the removal of 228 duplicates, 1,098 records were kept for screening. A total of 814 articles were excluded at the title and abstract review stage and 173 articles were excluded at the full-text review stage resulting in 62 articles selected for data extraction.

**Figure 2:**
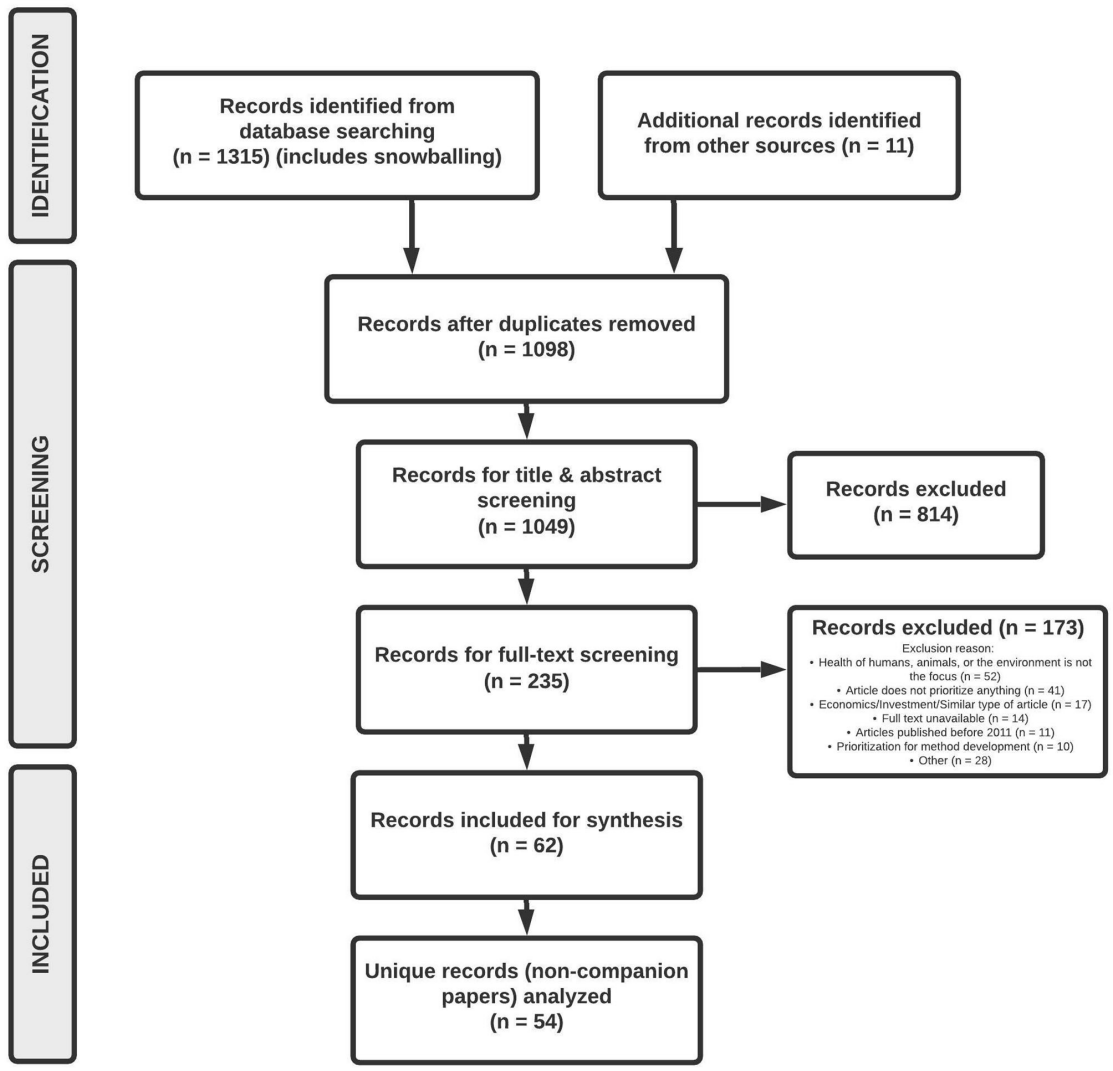
Identification and screening of articles following the PRISMA flow diagram.

Out of the 62 articles selected for data extraction and synthesis, eight were identified as a companion paper. Companion papers were those for which the findings of one prioritization study were published across multiple papers. The data from companion papers were combined with the primary paper and counted as one record resulting in a total of 54 studies examined for the purpose of this review. A PRISMA flowchart of the literature search is shown in Figure 2.

A Kappa score of 0.40 was obtained for inter-rater agreement during the title and abstract screening phase and the corresponding Cohen's Kappa score interpretation of 0.40 is “fair” (10). This Kappa score consisted only of articles identified via an initial search for articles in online databases and did not include articles from the revised search or gray literature. Following a review of articles in which disagreements arose, articles were either included for full-text screening or excluded based on consensus between the two reviewers. While a Kappa score was not calculated for full-text screening, the two reviewers discussed articles in which disagreements arose and similarly included or excluded articles for synthesis based on consensus.

### General Context

A consistent presence of studies in the literature was observed over the time span covered, with at least two articles published each year between 2010 and April 2021 (Table 1). The most prominent OECD member countries featured in published articles were Canada (*n* = 11; 20%), the United Kingdom (*n* = 4; 7%), and Turkey (*n* = 4; 7%). Several articles were based on multiple countries, most commonly members of the European Union (*n* = 5; 9%). An overwhelming majority of articles (*n* = 37; 69%) resided in the human domain (Table 1). A considerable number of articles in the animal (*n* = 9; 17%) and environmental (*n* = 6; 11%) were identified, and a small number of articles covered both the human and animal domains (*n* = 2; 4%).

**Table 1:** Summary of article characteristics.

**Characteristic**	**Articles *N* (%)**
**Publication year**	
2010	6 (10%)
2011	3 (5%)
2012	7 (11%)
2013	7 (11%)
2014	5 (8%)
2015	7 (11%)
2016	6 (10%)
2017	4 (6%)
2018	5 (8%)
2019	2 (3%)
2020	8 (13%)
2021	2 (3%)
**Country of focus**	
Canada	11 (20%)
Turkey	4 (7%)
United Kingdom	4 (7%)
Australia	3 (6%)
Switzerland	3 (6%)
Belgium	2 (4%)
Germany	2 (4%)
Greece	2 (4%)
Japan	2 (4%)
Netherlands	2 (4%)
Norway	2 (4%)
United States	2 (4%)
Sweden	1 (2%)
Chile	1 (2%)
France	1 (2%)
Italy	1 (2%)
New Zealand	1 (2%)
Multiple countries	10 (19%)
European Union	5 (9%)
International	2 (4%)
North America	1 (2%)
Canada and Burkino Faso	1 (2%)
Netherlands and Slovakia	1 (2%)
**Domain**	
Human	37 (69%)
Animal	9 (17%)
Environment	6 (11%)
Animal + Human	2 (4%)

### Prioritization Context

Of the 54 Primary Articles Analyzed, 28 (52%) Prioritized Threats to Human, Animal, and Environmental health and 23 (43%) prioritized interventions to manage threats to the health of these domains (Table 1). Other categories included prioritizing both threats and interventions (*n* = 1), prioritizing geographic areas by population vulnerability (*n* = 1), and prioritizing criteria used in tool development for assessing health technologies (*n* = 1).

Most articles used prioritization to inform decisions in disease management (*n* = 33; 61%) (e.g., resource allocation). This decision category was exclusive to the human and animal domains, at 62 and 89% of studies, respectively. In terms of topic area, the majority of articles focused on prioritization related to infectious diseases (*n* = 33; 61%), which was also driven by studies in the human (62%) and animal (100%) domains. Studies within the environmental domain prioritized either environmental hazard interventions (*n* = 5) or ecosystem management strategies to maintain forest health (*n* = 1).

**Figure 3:**
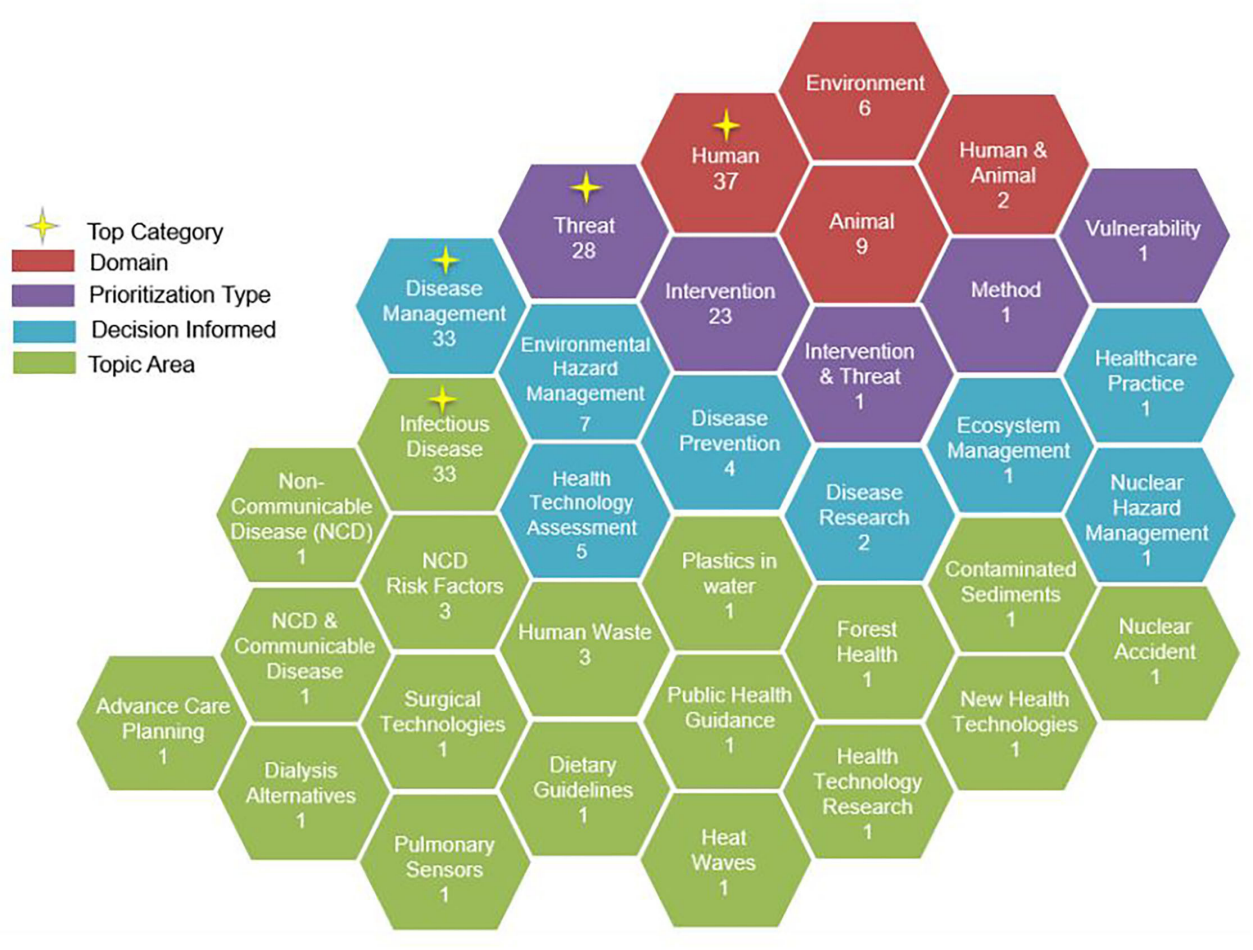
Overview of study contexts by domain, decisions informed, prioritization type, and topic area.

The objectives of studies examined fell into two broad categories: (a) method development and testing and (b) informing health-related policies or practices. Method development and testing objectives were the primary focus in 31 (57%) articles and included developing a framework/tool, assessing a particular method, or comparing methods used in different contexts. These studies were typically conducted through pilot exercises or by expanding on previous work. On the other hand, informing health-related policies or practices was the focus of 23 (43%) articles. This included studies that ranked different health initiatives to obtain scores or a prioritization list that could be used to inform decision-making. A visualization of study contexts by domain, decisions informed, priortization type, and topic area can be seen in Figure 3.

### Decision Support Method and Approach

A vast majority of studies used MCDA as the decision support method (*n* = 53; 98%). The one non-MCDA study involved the prioritization of criteria via the Delphi technique. Most articles (*n* = 40; 74%) used a value-based MCDA approach in which an overall score was calculated based on the weighted average of the criteria. The second most common method was an outranking approach (*n* = 9; 17%) in which an aggregate score was generated through a pairwise comparison of the alternatives. Two studies employed a reference-based MCDA approach in which different alternatives were rated relative to a gold standard. Two studies employed mixed methods. Studies in the human and animal domain primarily used value-based MCDA, at 81 and 89% of studies, respectively. Studies in the environmental domain were evenly split across the value-based (*n* = 2), outranking (*n* = 2), and reference based (*n* = 2) methods and were the only studies using the reference-based approach in our review.

Most studies (*n* = 37; 69%) conducted uncertainty analysis. There was considerable variation in the methods employed, and just under half of these studies used more than one method (*n* = 16). The most common methods included varying weights (*n* = 21) and criteria scores (*n* = 12) to assess rank-order stability. Other methods included using multiple weighting schemes, fuzzy analysis techniques, mathematical modeling to assess uncertainty *via* probability distributions, and removing certain criteria or stakeholder groups from the analysis. Uncertainty analysis methods did not appear to be influenced by domain.

Scoring attributes tended to cover complex mathematical equations requiring specialized subject matter expertise for their evaluation, resulting in their omission for the scope of this review.

### Criteria Analysis

**Figure 4:**
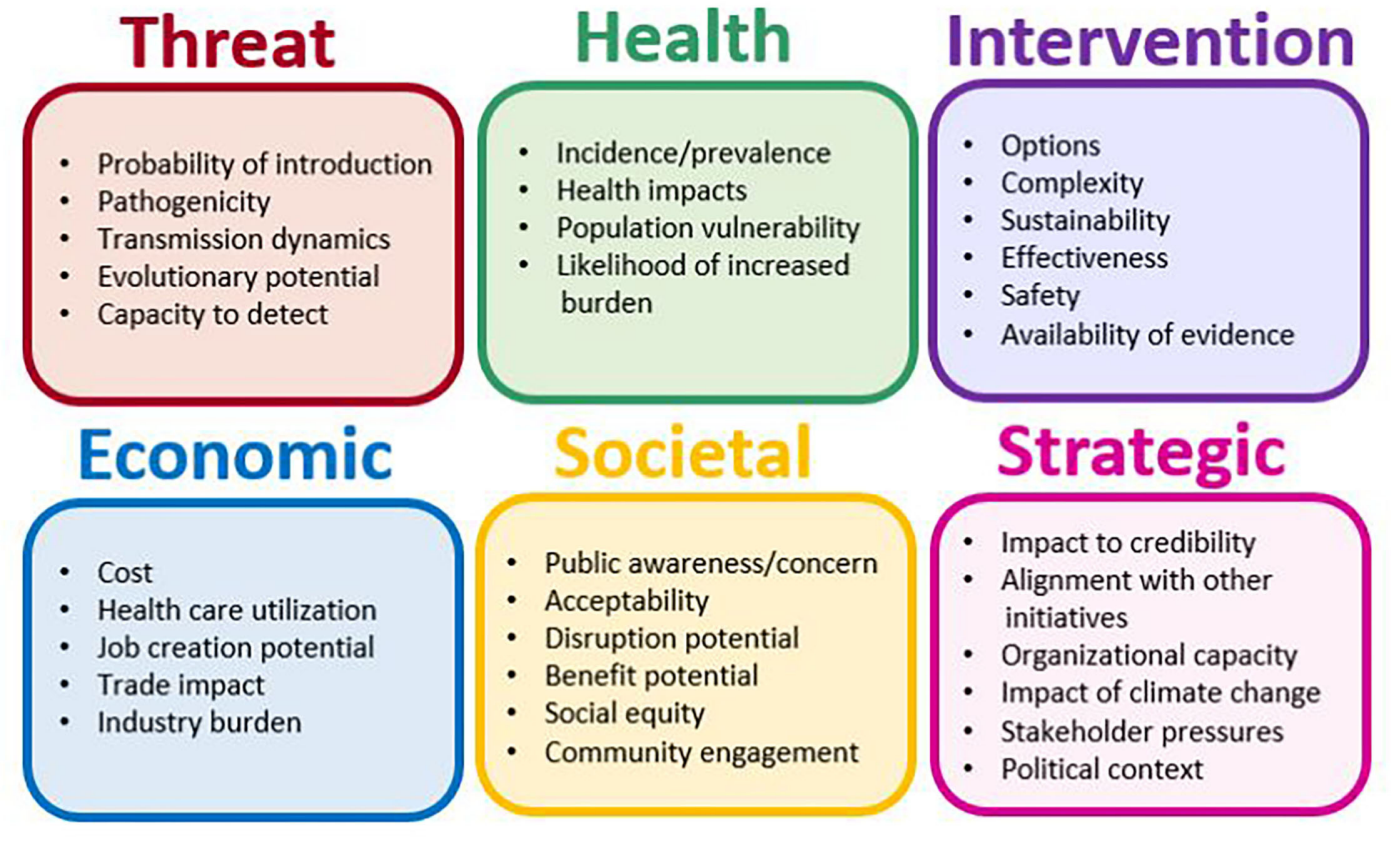
Examples of criteria and criteria groupings within each criteria category.

Criteria were typically selected based on reviews of the literature and, in many studies (*n* = 34; 63%), finalized through stakeholder engagement. The median number of criteria was 10, and the interquartile range was 8–18 criteria (range: 4–135). We observed hundreds of specific criteria, which fell into six main categories: nature of the threat, the relationship of the threat to human, animal or environmental health, details related to interventions, economic considerations, societal implications, and strategic factors (Figure 4). Criteria within these categories included measures of likelihood (e.g., disruption potential), impact (e.g., health impacts), vulnerability (e.g., vulnerable groups), and relevant context (e.g., available evidence). Many criteria covered similar concepts but used different wording or level of specificity. For example, some studies used “disease burden” while others used a combination of criteria to capture burden such as “incidence,” “case fatality rate,” “severity,” “chronicity,” and “health care utilization.” Across the human, animal, and environmental domains, the most common criteria used were those related to the impacts of the initiatives prioritized, particularly health, financial, and operational.

Criteria weighting occurred predominantly through direct methods (53%), wherein authors or stakeholders assigned values (e.g., allocating 100 points across all criteria). Comparison methods were also used frequently (36%), wherein criteria weights were obtained indirectly via comparative exercises (e.g., analytical hierarchy process). Other methods included the use of mathematical modeling and mixed methods. The weighting methods did not appear to be influenced by domain.

Where indicated, criteria performance assessment occurred by authors using literature and/or available data (37%), via stakeholders (28%) or a combination of both (35%). The majority (60%) evaluated criteria performance using measurement scales, whereby assessors selected among quantitative or qualitative bins to rate an alternative for given criteria (e.g., case fatality <5% or “low”). Other criteria evaluation approaches include model-derived ratings, estimates generated using pairwise comparison of criteria (e.g., analytical hierarchy process), and points estimates from the literature data analysis. Most studies in the human domain used measurement scales (68%). The approaches were more varied in the animal and environmental studies.

### Stakeholder Engagement

A vast majority of studies included stakeholder engagement (*n* = 46; 85%). Stakeholder engagement was common in the human (86%) and animal (100%) domain, and less in the environmental domain (50%). The most common stakeholder groups engaged included government (e.g., ministry/department reps, policy analysts etc.), non-governmental groups (e.g., advocacy groups, industry etc.), subject matter experts (e.g., economists, epidemiologists, doctors, nurses etc.) and the public (e.g., the general public, students, farmers etc.). Of those studies engaging stakeholders, most (52%) involved 30 stakeholders or less (range: 5–3,402). No trends were observed in the number of stakeholders engaged by domain. We observed stakeholder engagement across the MCDA process but most commonly at the criteria weighting step (Table 2). Stakeholders were engaged in various ways, including meetings, focus groups, one-on-one interviews, and email communication. While some engagements involved unstructured information sharing, others used surveys, the Delphi process, post-it note exercises, causal mapping, scenario ranking, nominal group technique, and pairwise comparison exercises to elicit stakeholder perspectives.

**Table 2:** Summary of the 54 published studies by study categories and characteristics published between 2010 and 2021 from OECD countries.

	**Study category (** ***N*** **)**			
**Study characteristic**	**All (54)**	**One health (13)**	**Disparate threat (7)**	**Cyclical (11)**
**Top prioritization topic**	**Infectious disease**	**Infectious disease**	**Non-Communicable disease**	**Infectious disease**
**Number of criteria**				
Median	10	16	6	10
Interquartile range	8–18	8–27	6–10	7–12
Range	4–135	4–57	4–35	4–39
**Study duration**, ***n*** **(%)**				
<6 months	7 (13)	0 (0)	1 (14)	2 (18)
6 months−1 year	5 (9)	3 (23)	0 (0)	1 (9)
1–2 years	8 (15)	2 (15)	0 (0)	2 (18)
>2 years	6 (11)	3 (23)	1 (14)	1 (9)
Not clearly defined	28 (52)	5 (39)	5 (71)	5 (45)
**Stakeholder engagement**, ***n*** **(%)**				
Set decision frame	10 (19)	3 (23)	1 (14)	2 (18)
Identify options	20 (37)	7 (54)	1 (14)	2 (18)
Select criteria	31 (57)	11 (85)	6 (86)	8 (73)
Weight criteria	41 (76)	13 (100)	6 (86)	8 (73)
Score criteria	28 (52)	6 (46)	5 (71)	7 (64)
Assess final ranking	9 (17)	4 (31)	2 (29)	4 (36)
Top method benefit	Analytic potential & usefulness			
Top method limitation	Persistence of bias and uncertainty			

Accessibility, when engaging with stakeholders, was a common theme highlighted in many papers. Given that stakeholders engaged often came from diverse backgrounds, it is essential that engagement strategies integrate methods to communicate effectively and efficiently. For example, the use of pairwise comparisons through discrete choice experiments was conducted via email to improve efficiency (11). Other ways to improve accessibility involved the use of a facilitator during workshop discussions (12). Questionnaires were also designed such that minimal prior knowledge of specialized topics was required (13). User-friendly interfaces for criteria scoring (14), data synthesis (15), and data exploration (16, 17) were also noted as helpful in improving the effectiveness of the stakeholder engagement process (18).

### Study Duration

We were unable to estimate study duration for over half of all articles (*n* = 28; 52%) due to a lack of information on time horizon provided within the article. Moreover, the intensity of work within the estimated duration was not described by authors. In other words, the amount of effort or hours invested into work on MCDAs on a day-by-day basis or within a defined time frame was ambiguous for the analyzed articles. For articles where study duration could be estimated (*n* = 26; 48%), most were <2 years: <6 months (*n* = 7; 13%), 6 months to 1 year (*n* = 5; 9%), 1–2 years (*n* = 8; 15%), and >2 years (*n* = 6; 11%).

### MCDA Strengths and Limitations

In terms of limitations, 17 (31%) studies highlighted that the MCDA model did not eliminate subjectivity of participants. Most notably, subjectivity was still present in the selection, weighting, and scoring of criteria. Examples of potential reasons for subjectivity include the background of stakeholders, influence from recent events, and overall use of expert opinion. However, it was also noted that MCDA provided the analytic potential to assess the impact of this bias via sensitivity analysis. Other forms of bias included selection bias (e.g., survey participation) and recall bias (e.g., rating avian influenza high following a pandemic). Twelve (23%) studies noted limited availability of evidence as a constraint and others noted the persistence of uncertainty. Although data availability and uncertainty are not unique to MCDA, these comments underscored another key point raised by many authors, summarized by Ruzante et al. “while MCDA methodologies provide tools to improve the decision-making process, they do not replace decision makers” (15). Authors noted that results were specific to the evidence available at the time of analysis and the perceptions of those selecting, weighting, and scoring the criteria. However, many studies also found the results to be robust through sensitivity analysis and comparable to findings of other studies. Another important finding to highlight is that 8 (15%) studies noted that MCDA was demanding (i.e., mentally exhausting for stakeholders and researchers involved) and time-consuming.

In terms of methodological strengths, 25 (46%) studies specifically noted that MCDA was beneficial to the decision-making process. Even among those studies wherein usefulness was not explicitly stated, the number of benefits highlighted by authors outweighed the limitations. MCDA was often described as a systematic, transparent, and flexible method that supports decision-making. Specifically, it was depicted as an effective focal point for collaboration by fostering evidence-based and structured multi-disciplinary deliberation and knowledge exchange. The criteria and scoring frameworks developed for specific studies were often characterized as reusable (e.g., could be updated over time), and found to be applicable in different geographic locations or similar decision requirement contexts. MCDA was seen as a way to simplify the “difficult process of managing decisions in complex scenarios” (19) and create the opportunity to “balance between comprehensiveness and simplicity” (20). One of the most recognized benefits of MCDA was its flexible and systematic approach. More specifically, the ability to incorporate quantitative and qualitative data, the ability to use multiple criteria to capture a more holistic assessment of risk/benefit, and the ability to examine the influence of different scenarios, criteria, and stakeholder groups on prioritization results.

### One Health

**Figure 5:**
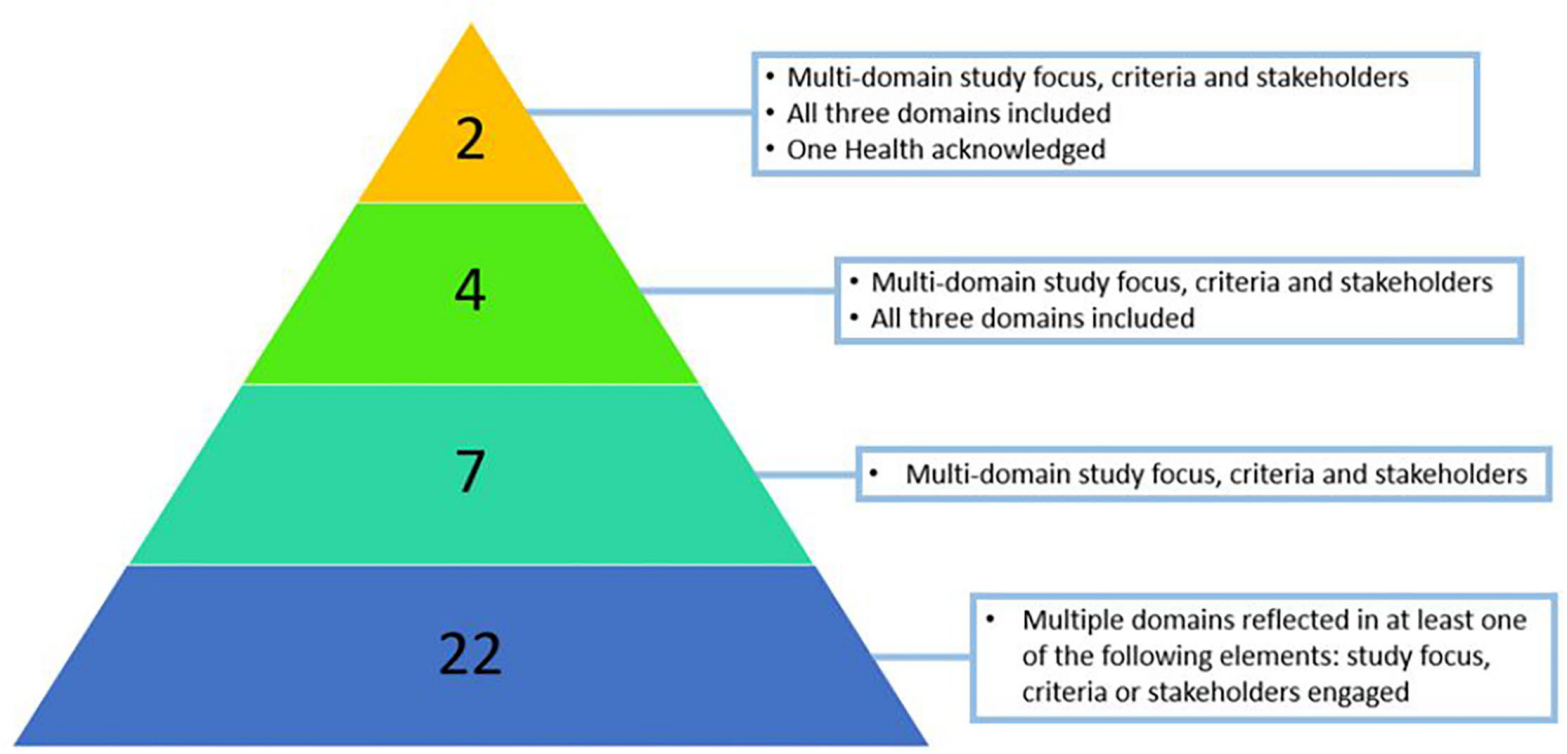
Number of studies at each level of the One Health definition.

Most articles (*n* = 35; 65%) included elements of our One Health definition, but only five studies (9%) explicitly acknowledged One Health as a driver for the study (14, 21–24). Among those studies meeting our definition, the application of the approach varied in terms of the depth of integration (Figure 5). Most studies (*n* = 21; 60%) included only one or two elements of our definition, typically the inclusion of criteria from another domain (e.g., one criterion related to human health impact in an environmental study). Seven (20%) studies included all three elements of our definition (14, 17, 24–28). Fewer studies (*n* = 4; 11%) met all three elements of our definition *and* included criteria or stakeholders from all three domains (18, 29–31). Only 2 studies (6%) met our One Health definition, reflected on all three domains, and acknowledged One Health as a driver for the study design (21, 22).

Our sub-study analysis included only those studies meeting all three elements of the One Health definition (*n* = 13). Most of these studies (*n* = 11; 85%) were on the topic of infectious disease directly influenced by other domains (e.g., zoonoses and vector-borne diseases). Where study duration information was available (*n*= 8), all lasted longer than 6 months. The median number of criteria used was 16, and the interquartile range was 8–27 criteria (range: 4–57). No notable difference was observed in the criteria included beyond the previously mentioned multi-domain aspect. In general, stakeholder engagement was greater across all process steps relative to all studies combined (Table 2). In addition, engaging stakeholders to assess the final ranking was rare (*n* = 9), and almost one-half of these were One Health studies. In terms of benefits and limitations, MCDA was noted to be “well-aligned with a One Health approach” (22) given the “potential of providing a holistic view that integrates conflicting criteria” (25) and the “development of a cross-disciplinary network” (26) for deliberation. Almost one-third of these studies (*n* = 4; 31%) noted that the method was time-consuming. One Health studies accounted for ~44% of all studies highlighting the time-consuming nature of MCDA.

### Disparate Threats

Seven studies were included in our disparate threat sub-analysis. Only one study prioritized disparate threats as per our definition by prioritizing both communicable and non-communicable diseases (32). Three studies prioritized interventions related to both communicable and non-communicable diseases (11, 33, 34). An additional three studies prioritized disparate public health interventions (e.g., environmental, therapeutic, behavioral) paired with distinct non-communicable disease threats (12, 13, 35). The prioritization topics included health technologies, public health guidance and interventions for non-communicable disease risk factor prevention. The criteria used in disparate threat studies were generally high level and broad. For example, instead of disease incidence and severity, studies used “disease burden” (32–34) or “size of the problem” (12). The criteria tended to consider the wide-ranging impacts of a threat or intervention at the societal level. This included the use of criteria such as “making a difference” (12), “unmet needs” as a measure of equity in access to care (32), impact on “wellbeing” (13), “distribution of benefit” (11), or “Ethical, legal, or psychosocial implications” (34).

In disparate threat studies, stakeholder engagement was centered on the criteria-based steps (i.e., selection, weighting, and scoring) and was higher at all three steps when compared to all studies (Table 2). As with other studies, the most identified limitation was the persistence of bias and uncertainty. No study specifically acknowledged that they prioritized alternatives that were disparate or difficult to compare relative to other studies. Athanasakis et al. did note, however, “criteria selection for the decision problem under scrutiny was a challenging exercise” (32). In addition, two studies noted that the allotted time for workshops was insufficient to meet stakeholder engagement objectives (12, 35). This subset of studies observed a key theme of thinking at the margin to balance the comprehensiveness of discussions and limited time. Howard et al. noted the value of out-of-session discussions to balance productive deliberations (33). Other strategies for achieving this balance included limiting the amount of time to discuss each topic during workshop sessions (13), limiting the amount of additional information requests from stakeholders (34), or performing extensive preparatory work on the selection and structuring of criteria prior to conducting conference workshops (32).

### Cyclical Prioritization for Governing Bodies

Eleven studies involved the development of MCDA tools recommended for recurrent cycles of evaluation and prioritization to inform decision-making processes by governing end-users (6, 12, 14, 15, 20, 33, 34, 36–39). Governing end-users included committees, institutes, organizations, and government officials. Most studies were conducted at the national level (*n* = 8; 73%) but also at state (*n* = 1; 9%), continental (*n* = 1; 9%) and international levels (*n* = 1; 9%). Three studies reflected on results from multiple cycles of varying lengths, including monthly (20), biannual (34), and annual (14). One study noted the intention to re-assess priorities within 5 years (36). Most studies involved the prioritization of infectious diseases (*n* = 8; 73%). The remaining three studies focused on health technologies (*n* = 2; 18%) and public health guidance (*n* = 1; 9%), all of which met the disparate threat definition. Study duration was available for over half of the articles and was distributed relatively evenly across the different time categories (Table 2). The median number of criteria was 10 with an interquartile range of 7–12 criteria (range: 4–39). No notable trend in criteria used for prioritization was observed in this subset of studies.

Stakeholder engagement was centered on criteria selection, weighting, and scoring. The proportion of studies engaging stakeholders at criteria selection, criteria scoring, and final rank assessment steps were greater in this subset of studies relative to all studies. Almost one-half of all One Health studies that engaged stakeholders to assess the final ranking involved cyclical prioritization for governing bodies. In total, seven of the nine studies that engaged stakeholders at this step were either One Health, cyclical prioritization studies or disparate threat studies. Similar benefits and limitations to other studies were noted. A recurring theme of transparent and systematic deliberative processes was present in these studies- emphasizing accountability and auditability of the prioritization process. A unique method to balance time vs. comprehensiveness during cyclical prioritization exercises was conducted by Kadohira et al. where starting times were staggered for different stakeholder groups (38). More traditional methods to manage time limitations were also used in this group of articles including limiting the scope of prioritization and using value-based analytical hierarchy approaches (12, 37).

A common pattern for stakeholder engagement approach involved re-using previously formed stakeholder groups or committees (6, 14, 33, 34, 36). Mehand et al. noted the importance of regularly changing committee members to avoid bias related to expert opinions (14). They also noted the value of having separate committees for the development and implementation of the method.

## Discussion

### Main Findings

Our analysis observed that the methods used in MCDA models and other similar decision-making support tools come in a patchwork of combinations. We observed variation across studies in the stakeholder groups engaged, criteria identification and weighting methods, intended targeted audiences, threats prioritized, and integration of One Health principles. In concurrence with other recent published literature reviews, our study found a sizable number of studies on the topics of pandemics and emerging diseases caused by climate change (40). Our analyses also highlight the inclusion of social and economic criteria, a research gap previously identified by Alsalem et al. (41). In the context of the broader knowledge base on the use of multi-criteria models to support decision making, our literature review is the first review (to our knowledge) that integrates a One Health lens for the prioritization of disparate health risks, threats, and interventions.

The primary objective of this paper was to identify key considerations for the application of a One Health based MCDA for all-hazard threat prioritization at the national level. By examining how multi-criteria decision-making support methods have been used in the human, animal, and environmental domains, we identified six important considerations through this analysis. A discussion follows.

### Multi-Criteria Decision Support Methods Are Useful to Support Complex Decision-Making and Can Enhance Collaboration

One of the most apparent findings of this review was the usefulness of employing an MCDA approach to support complex decision-making. Limitations were noted in each study regarding the effect of various factors on rank stability and representation of stakeholder values. However, the benefits of using the method far outweighed limitations for reasons that extended beyond the “right answer.” The value of MCDA was in the framework established for collaboration. MCDA has the added benefit of allowing collaborative efforts at each step of the analysis process. Stakeholders, which typically do not work together, can be involved in the MCDA process with minimal barriers to participation due to its transparent nature (42, 43). Moreover, MCDA offers the flexibility of utilizing various tools to facilitate stakeholders like surveys, workshops, or a Delphi technique. Finally, stakeholders can be selective in the parts of the MCDA process they influence. These findings coincide with previous literature by Drake et al. who concluded that the primary benefits of MCDA are the transparent, structured, and participatory aspects which support collaboration amongst stakeholders (44).

Collaboration is challenging, especially when it involves diverse stakeholders with competing interests. Decision-making on complex multifactorial problems is also challenging. Conflict and tension are inevitable in these scenarios. MCDA structures collaboration and evidence synthesis in a way that can minimize conflict and foster meaningful deliberation that can ultimately lead to synergistic knowledge exchange. This can be achieved when a shared understanding emerges through collaborative problem structuring, selection of options, and criteria. MCDA has the flexibility to accommodate diverse perspectives by incorporating various criteria. Engaging stakeholders in criteria weighting provides an opportunity for them to express their values. The synthesis of evidence required to conduct the MCDA and the ability to explore the impact of different criteria and weighting schemes creates the opportunity to explore an issue in more depth collaboratively. The resulting transparency of value trade-offs made to identify top priorities helps maintain social capital and helps achieve consensus.

### There Exists a Need for Additional Guidance on MCDA Method Selection and Guidance on Conducting MCDAs

MCDA was the most common method of prioritization of health initiatives, with an assortment of approaches including outranking, value-based, and reference-based methods. We also observed a wide range of methods employed at each step of the MCDA process. The use of these methods can be described as patchwork, where different methods within each category can be combined and mixed with other categories. It is known that each method has advantages and disadvantages in terms of complexity, time requirement, or accessibility to non-experts (5). It is also understood that methods chosen for a particular study may depend on the primary study objectives, purpose, and decision-maker needs and preferences (45). However, it was difficult to thematically extract method selection processes and justify using one method over another from the papers analyzed. No clear rules emerged, and the description of how the methods selected aligned with study objectives was rare. In most studies reviewed, it was challenging to discern the rationale for selecting MCDA type (i.e., value-based vs. outranking) and criteria measurement scales. In addition, we were unable to assess the impact of study duration requirements on method selection due to a lack of data (e.g., limiting criteria or stakeholders engaged).

The lack of justification for methods used has been observed in other reviews (46). In a recent critical analysis of multi-criteria models prioritizing health initiatives, Montibeller et al. advocated for careful design of prioritization models. They noted that many papers in their review lacked a “clear conceptual framework rooted on Multi-Criteria Decision Analysis” (47). Cinelli et al. recently published a taxonomy to support the application of MCDA (48). They noted the challenge in selecting the appropriate method and emphasized that there are consequences to selecting an inadequate approach, most notably a recommendation misaligned with the problem or stakeholder needs. Del Rio Vilas et al. recommended workshops be facilitated by an MCDA expert to prevent method errors (20). For researchers new to this analysis area, it is advisable to engage with method experts in the problem-structuring phase. Having a clear view of the intended decision required, the decision-maker's objectives, and the decision-making context is essential to inform method selection and study design. At a minimum, a clear understanding of the strengths and limitations of the analysis concerning the prioritization objectives will support constructive deliberations with stakeholders.

### Inclusion of Multiple Health Domains and Other One Health Elements in the Study Design Can Be Helpful but Is Not Essential

We did not observe differences in methods between domains that resulted in a specific key consideration. However, the analysis and our understanding of MCDA was enriched by looking at studies in different domains. While the animal and human domains were usually connected in terms of health impacts, articles in the environmental domain tended to be isolated from the other two domains. Specifically, environmental studies rarely considered impacts on human health or animal health. Instead, these studies focused on social or economic impacts resulting from risks or threats to environmental health. To deal with uncertainty, environmental studies also used different analysis methods, including reference-based and fuzzy analysis techniques. Studies in the animal domain contributed to our assessment of One Health and cyclical prioritization.

### The Integration of One Health Principles Approaches Are Not Standardized and May Increase Study Duration

The inclusion of criteria or stakeholders from different domains was common. However, a more rigorous approach to applying the principles of One Health was rare. Ultimately, there was no previous definition we were aware of in terms of what was required for a study to be considered a One Health MCDA. We hypothesize that this is because One Health principles are rarely explicitly considered in past prioritization studies. Thus, we constructed our own definition for our analysis^1^. Based on our classification approach, One Health studies had on average more criteria and, based on limited data, may have been longer in duration. This is plausible given multi-disciplinary collaboration across diverse fields would be more challenging. Research teams seeking to use a One Health approach will need to allot time in study design to consider what using a One Health approach means for them. It would be of value to share the outcome of that consideration with other researchers. The use of the MCDA methodology may help support the additional complexity of a One Health approach to prioritization. In line with the benefits of more effective knowledge integration for policy formulation as identified by Hitziger et al. (49) we recommend integrating One Health principles more systematically in future health prioritization exercises. However, it may be advisable to use simple criteria understandable to a wide range of stakeholders. In addition, research teams may need to add additional time to account for more criteria and the deliberation of experts from diverse fields.

### Prioritization of Disparate Threats Was Rare and Entailed Unique Method Considerations

The prioritization of disparate threats extended only as far as communicable vs. non-communicable disease by our definition, and only one study did this. One could argue that infectious diseases of different transmission routes are disparate or that communicable vs. non-communicable diseases are not disparate because they both involve disease. However, the key consideration for researchers seeking to prioritize threats in an all-hazard context is that there does not appear to be a large body of work to draw from for the prioritization of truly disparate threats (e.g., earthquake vs. influenza pandemic). The prioritization of disparate threats will impact the criteria selection. Minimal high-level criteria are more common when comparing disparate threats. Moreover, stakeholder discussions on disparate threats can be time-consuming and challenging given the different terminology and context within different fields.

Of note, no study was classified as both disparate and One Health. Combining these two approaches may be difficult when balancing comprehensiveness and potential for practical use in supporting decision-making. One limiting factor in integrating a One Health approach to disparate studies may be time; multiple articles limited the number of criteria or time spent on criteria discussions to balance comprehensiveness and time constraints (12, 20, 33). Disparate threats generally required fewer high-level criteria relative to One Health Studies (23). Moreover, the stakeholder engagement may be more complex given the various layers of expertise required to cover the different threats and the expertise related to human, animal, and environment dimensions of those threats.

### Cyclical Prioritization Entailed Unique Method Considerations

Cyclical prioritization involves the development of a reusable MCDA framework wherein the set of alternatives and the stakeholders engaged can change over time. Choosing to prioritize on an ongoing basis with a set of alternatives that can evolve over time entails unique considerations for the method development phase. If the set of alternatives are changing, it is recommended that an independent assessment of each alternative is preferable to a relative assessment (48). In this way, a reference-based approach may be of interest for cyclical prioritization, given the stability in the reference point. Mehand et al. indicated that their prioritization method was reviewed every 2 years and noted the use of different committees for method development and prioritization to reduce bias in the weighting step (14). The theme of accountability and auditability has implications for record-keeping and the development of user-friendly tools for sharing results. To inform government end users regularly, cyclical prioritization methods must be flexible enough to accommodate shifts in key MCDA components such as criteria used or stakeholder composition. An example of how to achieve this balance is the development of a user-friendly tool that is value-based, has a small set of core criteria, and is adaptable for multiple, diverse scenarios like the ones described in papers by Saito et al. (50) or Asselt et al. (17).

#### Strengths and Limitations of the Review

One benefit of our study is that we fill a gap in the current literature knowledge base on the use of MCDA and other similar decision support tools in public health decision-making through a One Health lens. Given that the concept of One Health has recently been in the academic and media spotlight because of recent events like SARS-CoV-2, we believe that our study will be highly beneficial by providing the groundwork for subsequent studies on similar topics.

A key strength of our study was the use of two reviewers and resolving conflicts through discussions to improve quality during article screening, data extraction and data analysis. We completed a thorough synthesis of the literature through both quantitative and qualitative analysis. Quantitative analysis was important in summarizing the literature identified such as the number of articles by publication year or the percentage of methods used. Thematic analysis provided a detailed examination of the reasons behind the use of various methods and their associated strengths and limitations.

One limitation of our literature search was conducting a comprehensive search of decision support tools and methodologies due to the vast search terms available. The number of synonyms for terms like multi-criteria analysis was significant and thus impossible to capture comprehensively. Furthermore, for feasibility considerations, the gray literature search was favored toward potentially higher-quality sources such as government websites and results from only the first five pages of Google Scholar. We also recognize that by limiting studies to those published since 2010, articles meeting our inclusion criteria may have been missed; however, this was a suitable time frame given the interest in One Health approaches. Another potential limitation of this study was that, given the focus of the analysis on specific items of interest, it is difficult to make general recommendations on the conduct of decision support tools and methods. Thematic analysis of benefits and limitations was based on extracts of data from the discussion; as such, the estimates of articles noting specific benefits of limitations are approximate.

The calculated Cohen's Kappa score of 0.40 for title and abstract screening can be interpreted as “fair” (10). While a greater Kappa score for inter-rater agreement may have been achieved using additional training articles, it would have also required additional time to do so. Most disagreements arose for the inclusion or exclusion of articles in the realm of environmental health. One reason for this may be that there was a diverse and often weak connection to public health in this category of articles. Examples of these articles include topics on forestry, marine health, and waste management. A Cohen's Kappa score of 0.40 sits on the upper marginal boundary between the “fair” classification (0.21–0.40) and “moderate” classification (0.41–0.60) (10). Given the problematic nature of article screening for a diverse set of articles with varying strengths of connection to the primary research objectives, it may be sufficient to proceed past article screening with a lower Kappa score under the condition that both reviewers have established confidence in their inclusion or exclusion of articles.

## Conclusion

The prioritization of health threats and interventions using multi-criteria decision support methods is a broad field of study. However, studies applying a comprehensive One Health approach, prioritizing disparate threats, or conducting cyclical prioritizations for governing bodies was relatively sparse. The processes used to prioritize health initiatives followed a similar structure across topics and domains, but the methods at each prioritization step varied considerably. Studies involving a One Health approach, disparate threats, and cyclical prioritization included broader stakeholder engagement and specific criteria requirements which when taken together could add complexity and more time to study duration. Based on the current literature, MCDA appears to be a useful tool in supporting complex decision-making and has many methodological benefits for governing end-users. However, these review results underline the need for better guidance on the use of different MCDA methods depending on the prioritization objectives, and the need for guidance on how to integrate a One Health approach. The results of this review will inform the development of a pilot MCDA model for the prioritization of threats and vulnerabilities at the national level by the Public Health Agency of Canada.

## Data Availability Statement

The original contributions presented in the study are included in the article/Supplementary Material, further inquiries can be directed to the corresponding author.

## Author Contributions

JZ and TS carried out the literature review including collection of articles, screening of articles, data extract, data analysis, and synthesis of results. JZ wrote the first draft of the manuscript. TS revised the manuscript. CA, RC, AF, AJ, JS, ML, and BH read and suggested a subsequent round of revisions for the manuscript. JZ completed the final revision of the manuscript. All authors contributed to manuscript revisions, read, and approved the submission of the manuscript.

## Funding

The Public Health Agency of Canada (PHAC) provided funding for open access publication fees.

## Conflict of Interest

The authors declare that the research was conducted in the absence of any commercial or financial relationships that could be construed as a potential conflict of interest.

## Publisher's Note

All claims expressed in this article are solely those of the authors and do not necessarily represent those of their affiliated organizations, or those of the publisher, the editors and the reviewers. Any product that may be evaluated in this article, or claim that may be made by its manufacturer, is not guaranteed or endorsed by the publisher.

## Footnotes

^1^Studies are considered to use One Health principles if it included any element of an MCDA (e.g., methods, stakeholders, criteria, etc.) which involve two or more of the human, animal, or environment domains.
