# Supplementary material for: A Rapid Literature Review of Multi-Criteria Decision Support Methods in the Context of One Health for All-Hazards Threat Prioritization
Source: Front Public Health. 2022 Apr 15;10:861594. doi: 10.3389/fpubh.2022.861594 (PMC9051240; doi:10.3389/fpubh.2022.861594)
Supplement: Supplementary file 1 [file Data_Sheet_1.pdf]

frontiers

Supplementary Material

1 Initial search strategy

Limit by OECD countries, English and French language. Remove editorials.

Database(s): **Embase** 1974 to 2021 February 01
Search Strategy:

#	Searches	Results
1	exp *multicriteria decision analysis/	452
2	exp *decision support system/ or *"comparative risk assessment"/	12706
3	(MCDA or MCDM or MADM or MAUM or MAVT or DCE or CRA or AHP).tw,kw.	18022
4	((multicriteria or multi* criteria) adj3 decision?).tw,kw.	1982
5	(multi* attribute adj3 (utility or value or decision)).tw,kw.	509
6	(decision support adj4 (tool? or system?)).tw,kw.	13164
7	(multiutility or multi utility or discrete choice experiment* or analytical hierarchy process* or comparative risk?).tw,kw.	4522
8	or/1-7	42894
9	(priorit* or scor* or rank* or outrank* or order*).tw,kw.	3414563
10	(weight* adj3 criteri*).tw,kw.	2749
11	or/9-10	3416306
12	exp *public health/ or *population health/ or exp *environmental health/ or *animal health/ or *one health/ or *ecosystem health/ or exp *veterinary medicine/	114428
13	exp *environmental impact assessment/ or exp *health impact assessment/	7941
14	((public or population or ecosystem? or environment* or animal? or ecolog*) adj2 health).tw,kw.	366678
15	(one health or one medicine or veterinary medicine).tw,kw.	14772
16	((environ* or health) adj2 assess*).tw,kw.	72458
17	or/12-16	524715
18	and/8,11,17	714
19	editorial/ or editorial.pt. or editorial?.tw,kw.	772793
20	18 not 19	711

21	exp "Australia and New Zealand"/ or Australian/ or new Zealander/ or indigenous Australian/ or Austrian/ or Austria/ or Belgium/ or Belgian/ or exp Canada/ or Canadian/ or Canadian aboriginal/ or Chile/ or Chilean/ or Czech Republic/ or "czech (citizen)"/ or Denmark/ or Danish citizen/ or exp Baltic states/ or "estonian (citizen)"/ or Finland/ or "finn (citizen)"/ or France/ or frenchman/ or exp Germany/ or "german (citizen)"/ or Greece/ or "greek (citizen)"/ or Hungary/ or "hungarian (citizen)"/ or Iceland/ or Icelander/ or Ireland/ or "irish (citizen)"/ or Israel/ or israeli/ or Italy/ or "italian (citizen)"/ or Japan/ or "japanese (citizen)"/ or south korea/ or south korean/ or "latvian (citizen)"/ or Lithuania/ or "Lithuanian (citizen)"/ or Luxembourg/ or Mexico/ or mexican/ or Netherlands/ or dutchman/ or exp Norway/ or "norwegian (citizen)"/ or Poland/ or polish citizen/ or Portugal/ or "portuguese (citizen)"/ or Slovakia/ or "slovak (citizen)"/ or Slovenia/ or "slovenian (citizen)"/ or Spain/ or Spaniard/ or Sweden/ or swedish citizen/ or Switzerland/ or swiss/ or "Turkey (republic)"/ or Turkish citizen/ or exp United Kingdom/ or exp british citizen/ or exp United States/ or american/ or american indian/ or alaska native/ or (australia* or new south wales or queensland or tasmania or victoria or sydney or melbourne or brisbane or adelaide or austria* or vienna or viennese* or belgium* or belgian* or brussels or flemish* or canad* or ottawa* or british columbia* or colombie britannique* or vancouver* or alberta* or edmonton* or calgar* or saskatchewan* or regina* or saskatoon* or manitoba* or winnipeg* or ontari* or toronto* or quebec* or montreal* or new brunswick* or nouveau brunswick* or fredericton* or nova scotia* or nouvelle ecosse* or halifax* or haligonian* or prince edward island* or ile du prince edouard* or pei or charlottetown* or newfoundland* or terre neuve* or labrador* or nfld or yukon* or whitehorse* or northwest territor* or territoires du nord ouest* or nwt or yellowknife* or nunavut* or iqaluit* or chile* or santiago or czech* or prague or denmark* or danish or dane* or faroe* or copenhagen or estonia* or tallinn or finland* or finnish* or helsinki* or france* or french* or paris* or marseille or lyon or lille or nice or toulouse or bordeaux or german* or deutschland* or berlin* or hamburg or munich or cologne or frankfurt or stuttgart or dusseldorf or greece* or hellenic* or greek* or athens or macedonia* or hungary* or hungarian* or budapest or iceland* or reykjavik or ireland* or irish* or dublin* or israel* or jerusalem or tel aviv or italy or italian* or rome or milan or naples or turin or sicily or japan* or tokyo or yokohama or osaka or nagoya or sapporo or kobe or kyoto or korea* or seoul or busan or daegu or daejeon or gwangju or incheon or ulsan or latvia* or riga or lithuania* or vilnius or luxembourg* or netherland* or holland* or dutch* or amsterdam or rotterdam or hague or new zealand* or aotearoa	6024473

	or wellington or auckland or maori or mexic* or norway* or norwegian* or oslo or poland* or polish or warsaw or krakow or wroclaw or lodz or portug* or lisbon or slovak* or bratislava or slovenia* or slovene* or ljubljana or spain* or spanish* or spaniard* or madrid or barcelona or catalonia* or valencia* or seville or zaragoza or malaga or basque or scandinavia* or sweden or swedish or swede* or stockholm or switzerland* or swiss* or zurich or geneva or bern or turkey or turkish or istanbul or constantinople or britain* or british* or united kingdom* or scotland* or scottish or wales* or welsh or england* or belfast or london or manchester or glasgow or birmingham or leeds or bradford or liverpool or alabama* or alaska* or arizona* or arkansas* or california* or colorado* or connecticut* or delaware* or florida* or georgia* or hawaii* or idaho* or illinois* or indiana* or iowa* or kansas* or kentucky* or louisiana* or maine* or maryland* or massachusetts* or michigan* or minnesota* or mississippi* or missouri* or montana* or nebraska* or nevada* or new hampshire* or new jersey* or new mexico* or new york* or north carolina* or north dakota* or ohio* or oklahoma* or oregon* or pennsylvania* or rhode island* or south carolina* or south dakota* or tennessee* or texas* or utah* or vermont* or virginia* or washington* or west virginia* or wisconsin* or wyoming* or montgomery* or juneau* or anchorage* or phoenix* or little rock* or sacramento* or los angeles* or san diego* or san francisco* or denver* or hartford* or dover* or tallahassee* or miami* or orlando* or atlanta* or honolulu* or boise* or springfield* or chicago* or des moines* or topeka* or frankfort* or baton rouge* or new orleans* or augusta* or annapolis* or boston* or lansing* or detroit* or st?paul* or jackson* or jefferson city* or helena* or lincoln* or carson city* or reno* or las vegas* or concord* or trenton* or santa fe* or albany* or raleigh* or bismarck* or columbus* or oklahoma city* or salem* or harrisburg* or providence* or columbia* or peirre* or nashville* or austin* or dallas* or salt lake city* or montpelier* or richmond* or olympia* or seattle* or charleston* or madison* or cheyenne* or district of columbia* or usa or united states).tw,kw.	
22	20 and 21	291
23	limit 22 to (english or french)	283

Database(s): **Ovid MEDLINE(R) ALL** 1946 to February 01, 2021
Search Strategy:

#	Searches	Results
1	*decision support techniques/	12191

2	(MCDA or MCDM or MADM or MAUM or MAVT or DCE or CRA or AHP).tw,kw,kf.	11979
3	((multicriteria or multi* criteria) adj3 decision?).tw,kw,kf.	1472
4	(multi* attribute adj3 (utility or value or decision)).tw,kw,kf.	381
5	(decision support adj4 (tool? or system?)).tw,kw,kf.	9976
6	(multiutility or multi utility or discrete choice experiment* or analytical hierarchy process* or comparative risk?).tw,kw,kf.	3173
7	or/1-6	35718
8	(priorit* or scor* or rank* or outrank* or order*).tw,kw,kf.	2444374
9	(weight* adj3 criteri*).tw,kw,kf.	1892
10	or/8-9	2445584
11	*public health/ or exp *population health/ or exp *environmental health/ or *one health/ or exp *veterinary medicine/	104588
12	exp *environmental impact assessment/ or exp *health impact assessment/	500
13	((public or population or ecosystem? or environment* or animal? or ecolog*) adj2 health).tw,kw,kf.	344164
14	(one health or one medicine or veterinary medicine).tw,kw,kf.	14116
15	((environ* or health) adj2 assess*).tw,kw,kf.	53632
16	or/11-15	462795
17	and/7,10,16	587
18	editorial/ or editorial.pt. or editorial?.tw,kw.	585682
19	17 not 18	586
20	exp australia/ or austria/ or exp baltic states/ or exp belgium/ or exp canada/ or chile/ or czech republic/ or exp "scandinavian and nordic countries"/ or exp france/ or exp germany/ or greece/ or hungary/ or exp ireland/ or israel/ or exp italy/ or exp japan/ or exp republic of korea/ or luxembourg/ or mexico/ or exp netherlands/ or exp new zealand/ or poland/ or exp portugal/ or slovakia/ or slovenia/ or exp spain/ or exp switzerland/ or turkey/ or exp united kingdom/ or exp united states/ or (australia* or new south wales or queensland or tasmania or victoria or sydney or melbourne or brisbane or adelaide or austria* or vienna or viennese* or belgium* or belgian* or brussels or flemish* or canad* or ottawa* or british columbia* or colombie britannique* or vancouver* or alberta* or edmonton* or calgar* or saskatchewan* or regina* or saskatoon* or manitoba* or winnipeg* or ontari* or toronto* or quebec* or montreal* or new brunswick* or nouveau brunswick* or fredericton* or nova	4663766

	scotia* or nouvelle ecosse* or halifax* or haligonian* or prince edward island* or ile du prince edouard* or pei or charlottetown* or newfoundland* or terre neuve* or labrador* or nfld or yukon* or whitehorse* or northwest territor* or territoires du nord ouest* or nwt or yellowknife* or nunavut* or iqaluit* or chile* or santiago or czech* or prague or denmark* or danish or dane* or faroe* or copenhagen or estonia* or tallinn or finland* or finnish* or helsinki* or france* or french* or paris* or marseille or lyon or lille or nice or toulouse or bordeaux or german* or deutschland* or berlin* or hamburg or munich or cologne or frankfurt or stuttgart or dusseldorf or greece* or hellenic* or greek* or athens or macedonia* or hungary* or hungarian* or budapest or iceland* or reykjavik or ireland* or irish* or dublin* or israel* or jerusalem or tel aviv or italy or italian* or rome or milan or naples or turin or sicily or japan* or tokyo or yokohama or osaka or nagoya or sapporo or kobe or kyoto or korea* or seoul or busan or daegu or daejeon or gwangju or incheon or ulsan or latvia* or riga or lithuania* or vilnius or luxembourg* or netherland* or holland* or dutch* or amsterdam or rotterdam or hague or new zealand* or aotearoa or wellington or auckland or maori or mexic* or norway* or norwegian* or oslo or poland* or polish or warsaw or krakow or wroclaw or lodz or portug* or lisbon or slovak* or bratislava or slovenia* or slovene* or ljubljana or spain* or spanish* or spaniard* or madrid or barcelona or catalonia* or valencia* or seville or zaragoza or malaga or basque or scandinavia* or sweden or swedish or swede* or stockholm or switzerland* or swiss* or zurich or geneva or bern or turkey or turkish or istanbul or constantinople or britain* or british* or united kingdom* or scotland* or scottish or wales* or welsh or england* or belfast or london or manchester or glasgow or birmingham or leeds or bradford or liverpool or alabama* or alaska* or arizona* or arkansas* or california* or colorado* or connecticut* or delaware* or florida* or georgia* or hawaii* or idaho* or illinois* or indiana* or iowa* or kansas* or kentucky* or louisiana* or maine* or maryland* or massachusetts* or michigan* or minnesota* or mississippi* or missouri* or montana* or nebraska* or nevada* or new hampshire* or new jersey* or new mexico* or new york* or north carolina* or north dakota* or ohio* or oklahoma* or oregon* or pennsylvania* or rhode island* or south carolina* or south dakota* or tennessee* or texas* or utah* or vermont* or virginia* or washington* or west virginia* or wisconsin* or wyoming* or montgomery* or juneau* or anchorage* or phoenix* or little rock* or sacramento* or los angeles* or san diego* or san francisco* or denver* or hartford* or dover* or tallahassee* or miami* or orlando* or atlanta* or honolulu* or boise* or springfield* or chicago* or des moines* or topeka* or frankfort* or baton rouge* or new orleans* or augusta* or annapolis* or boston* or lansing* or detroit* or st?paul* or jackson*	

	or jefferson city* or helena* or lincoln* or carson city* or reno* or las vegas* or concord* or trenton* or santa fe* or albany* or raleigh* or bismarck* or columbus* or oklahoma city* or salem* or harrisburg* or providence* or columbia* or peirre* or nashville* or austin* or dallas* or salt lake city* or montpelier* or richmond* or olympia* or seattle* or charleston* or madison* or cheyenne* or district of columbia* or usa or united states).tw,kf.	
21	19 and 20	245
22	limit 21 to (english or french)	234

Database(s): **CAB Abstracts** 1973 to 2021 Week 04
Search Strategy:

#	Searches	Results
1	decision support systems/	2521
2	(MCDA or MCDM or MADM or MAUM or MAVT or DCE or CRA or AHP).tw.	5681
3	((multicriteria or multi* criteria) adj3 decision?).tw.	2244
4	(multi* attribute adj3 (utility or value or decision)).tw.	297
5	(decision support adj4 (tool? or system?)).tw.	8951
6	(multiutility or multi utility or discrete choice experiment* or analytical hierarchy process* or comparative risk?).tw.	2046
7	or/1-6	16664
8	(priorit* or scor* or rank* or outrank* or order*).tw.	843839
9	(weight* adj3 criteri*).tw.	1421
10	or/8-9	844600
11	public health/ or exp *environmental health/ or animal health/ or veterinary medicine/	116706
12	environmental assessment/ or exp health impact assessment/	19462
13	((public or population or ecosystem? or environment* or animal? or ecolog*) adj2 health).tw.	184491
14	(one health or one medicine or veterinary medicine).tw.	29918
15	((environ* or health) adj2 assess*).tw.	39537
16	or/11-15	243963
17	editorials/ or editorial.pt. or editorial?.tw.	5422
18	exp oecd countries/	2231757

19	(australia* or new south wales or queensland or tasmania or victoria or sydney or melbourne or brisbane or adelaide or austria* or vienna or viennese* or belgium* or belgian* or brussels or flemish* or canad* or ottawa* or british columbia* or colombie britannique* or vancouver* or alberta* or edmonton* or calgar* or saskatchewan* or regina* or saskatoon* or manitoba* or winnipeg* or ontari* or toronto* or quebec* or montreal* or new brunswick* or nouveau brunswick* or fredericton* or nova scotia* or nouvelle ecosse* or halifax* or haligonian* or prince edward island* or ile du prince edouard* or pei or charlottetown* or newfoundland* or terre neuve* or labrador* or nfld or yukon* or whitehorse* or northwest territor* or territoires du nord ouest* or nwt or yellowknife* or nunavut* or iqaluit* or chile* or santiago or czech* or prague or denmark* or danish or dane* or faroe* or copenhagen or estonia* or tallinn or finland* or finnish* or helsinki* or france* or french* or paris* or marseille or lyon or lille or nice or toulouse or bordeaux or german* or deutschland* or berlin* or hamburg or munich or cologne or frankfurt or stuttgart or dusseldorf or greece* or hellenic* or greek* or athens or macedonia* or hungary* or hungarian* or budapest or iceland* or reykjavik or ireland* or irish* or dublin* or israel* or jerusalem or tel aviv or italy or italian* or rome or milan or naples or turin or sicily or japan* or tokyo or yokohama or osaka or nagoya or sapporo or kobe or kyoto or korea* or seoul or busan or daegu or daejeon or gwangju or incheon or ulsan or latvia* or riga or lithuania* or vilnius or luxembourg* or netherland* or holland* or dutch* or amsterdam or rotterdam or hague or new zealand* or aotearoa or wellington or auckland or maori or mexic* or norway* or norwegian* or oslo or poland* or polish or warsaw or krakow or wroclaw or lodz or portug* or lisbon or slovak* or bratislava or slovenia* or slovene* or ljubljana or spain* or spanish* or spaniard* or madrid or barcelona or catalonia* or valencia* or seville or zaragoza or malaga or basque or scandinavia* or sweden or swedish or swede* or stockholm or switzerland* or swiss* or zurich or geneva or bern or turkey or turkish or istanbul or constantinople or britain* or british* or united kingdom* or scotland* or scottish or wales* or welsh or england* or belfast or london or manchester or glasgow or birmingham or leeds or bradford or liverpool or alabama* or alaska* or arizona* or arkansas* or california* or colorado* or connecticut* or delaware* or florida* or georgia* or hawaii* or idaho* or illinois* or indiana* or iowa* or kansas* or kentucky* or louisiana* or maine* or maryland* or massachusetts* or michigan* or minnesota* or mississippi* or missouri* or montana* or nebraska* or nevada* or new hampshire* or new jersey* or new mexico* or new york* or north carolina* or north dakota* or ohio* or oklahoma* or oregon* or pennsylvania* or rhode island* or south carolina* or south dakota* or	3079116

	tennessee* or texas* or utah* or vermont* or virginia* or washington* or west virginia* or wisconsin* or wyoming* or montgomery* or juneau* or anchorage* or phoenix* or little rock* or sacramento* or los angeles* or san diego* or san francisco* or denver* or hartford* or dover* or tallahassee* or miami* or orlando* or atlanta* or honolulu* or boise* or springfield* or chicago* or des moines* or topeka* or frankfort* or baton rouge* or new orleans* or augusta* or annapolis* or boston* or lansing* or detroit* or st?paul* or jackson* or jefferson city* or helena* or lincoln* or carson city* or reno* or las vegas* or concord* or trenton* or santa fe* or albany* or raleigh* or bismarck* or columbus* or oklahoma city* or salem* or harrisburg* or providence* or columbia* or peirre* or nashville* or austin* or dallas* or salt lake city* or montpelier* or richmond* or olympia* or seattle* or charleston* or madison* or cheyenne* or district of columbia* or usa or united states).tw.	
20	or/18-19	3080001
21	and/7,10,16	401
22	21 and 19	165
23	limit 22 to (english or french)	157

SCOPUS

1.1 (((TITLE-ABS-KEY (mcda OR mcdm OR madm OR maum OR mavt OR dce OR cra OR ahp)) OR (TITLE-ABS-KEY ((multicriteria OR "multi* criteria") W/3 decision?)) OR (TITLE-ABS-KEY ("multi* attribute" W/3 (utility OR value OR decision))) OR (TITLE-ABS-KEY ("decision support" W/4 (tool? OR system?))) OR (TITLE-ABS-KEY (multiutility OR "multi utility" OR "discrete choice experiment*" OR "analytical hierarchy process*" OR "comparative risk?"))) AND ((TITLE (scor* OR rank* OR outrank* OR order*)) OR (TITLE-ABS (priorit*)) OR (TITLE-ABS-KEY (weight* W/3 criteri*))) AND ((TITLE-ABS-KEY ((public OR population OR ecosystem? OR environment* OR animal? OR ecolog*) W/2 health)) OR (TITLE-ABS-KEY ("one health" OR "one medicine" OR "veterinary medicine")) OR (TITLE-ABS-KEY ((environ* OR health) W/2 assess*)))) AND NOT (TITLE-ABS-KEY (australia* OR austria* OR belgium OR belgian OR canada OR canadian OR chile OR czech OR denmark OR danish OR estonia OR finland OR finnish OR france OR french OR german* OR greece OR greek OR hungary OR hunarian OR iceland* OR ireland OR irish OR israel OR israeli OR italy OR italian OR japan* OR korea* OR latvia* OR luxembourg OR mexico OR mexican OR netherland OR dutch O

R "new zealand" OR nz OR "new
zealander" OR norway OR norwegian OR poland OR polish OR portugal OR portuguese OR slovakia OR slovak OR slovenia* OR spain OR spanish OR sweden OR swedish OR switzerland OR swiss OR turkey OR turkish OR britain OR british OR {united
kingdom} OR uk OR scotland OR scottish OR wales OR welsh OR england OR {northern ireland} OR {united states} OR usa OR american OR america)) AND NOT INDEX (medline) AND (EXCLUDE (DOCTYPE , "ed")) AND (LIMIT-TO (LANGUAGE , "English") OR LIMIT-TO (LANGUAGE , "French")) 272

2 Refined second search strategy

Limit by OECD countries, English and French language. Remove editorials.

Database(s): **Embase** 1974 to 2021 April 08 (MCDMEmbSmith2Rev3Final
Search Strategy:

#	Searches	Results
1	exp *multicriteria decision analysis/	494
2	exp *decision support system/ or *"comparative risk assessment"/	12962
3	(MCDA or MCDM or MADM or MAUM or MAVT or DCE or CRA or AHP).tw,kw.	18361
4	((multicriteria or multi* criteria) adj3 (decision? or approach* or model* or technique*)).tw,kw.	2413
5	(multi* attribute adj3 (utility or value or decision)).tw,kw.	527
6	(decision support adj4 (tool? or system?)).tw,kw.	13465
7	(multiutility or multi utility or discrete choice experiment* or analytical hierarchy process* or comparative risk? or conjoint analy*).tw,kw.	5709
8	or/1-7	45120
9	(priorit* or scor* or rank* or outrank* or order*).tw,kw.	3480157
10	(weight* adj3 criteri*).tw,kw.	2810
11	or/9-10	3481934
12	exp *public health/ or *population health/ or exp *environmental health/ or *animal health/ or *one health/ or *ecosystem health/ or exp *veterinary medicine/ or exp *climate change/	134399
13	communicable diseases/pc or *zoonoses/pc or *epidemic/pc	4877
14	exp *environmental impact assessment/ or exp *health impact assessment/	8061

15	(communicable disease? or infectious disease? or infectious animal disease? or outbreak? or zoonos* or climate change).ti,kw. or (communicable disease? or infectious disease? or infectious animal disease? or outbreak? or zoonos* or climate change).ab. /freq=2	138901
16	((public or population or ecosystem? or environment* or animal? or ecolog*) adj2 health).tw,kw.	376274
17	(one health or one medicine or veterinary medicine).tw,kw.	15232
18	((environ* or health) adj2 assess*).tw,kw.	74040
19	or/12-16	589646
20	and/8,11,19	571
21	Health Priorities/ or (health* adj2 priorit*).tw,kw.	103096
22	and/8,21	528
23	or/20,22	1026
24	editorial/ or editorial.pt. or editorial?.tw,kw.	782829
25	23 not 24	1020
26	exp "Australia and New Zealand"/ or Australian/ or new Zealander/ or indigenous Australian/ or Austrian/ or Austria/ or Belgium/ or Belgian/ or exp Canada/ or Canadian/ or Canadian aboriginal/ or Chile/ or Chilean/ or Czech Republic/ or "czech (citizen)"/ or Denmark/ or Danish citizen/ or exp Baltic states/ or "estonian (citizen)"/ or Finland/ or "finn (citizen)"/ or France/ or frenchman/ or exp Germany/ or "german (citizen)"/ or Greece/ or "greek (citizen)"/ or Hungary/ or "hungarian (citizen)"/ or Iceland/ or Icelander/ or Ireland/ or "irish (citizen)"/ or Israel/ or israeli/ or Italy/ or "italian (citizen)"/ or Japan/ or "japanese (citizen)"/ or south korea/ or south korean/ or "latvian (citizen)"/ or Lithuania/ or "Lithuanian (citizen)"/ or Luxembourg/ or Mexico/ or mexican/ or Netherlands/ or dutchman/ or exp Norway/ or "norwegian (citizen)"/ or Poland/ or polish citizen/ or Portugal/ or "portuguese (citizen)"/ or Slovakia/ or "slovak (citizen)"/ or Slovenia/ or "slovenian (citizen)"/ or Spain/ or Spaniard/ or Sweden/ or swedish citizen/ or Switzerland/ or swiss/ or "Turkey (republic)"/ or Turkish citizen/ or exp United Kingdom/ or exp british citizen/ or exp United States/ or american/ or american indian/ or alaska native/ or (australia* or new south wales or queensland or tasmania or victoria or sydney or melbourne or brisbane or adelaide or austria* or vienna or viennese* or belgium* or belgian* or brussels or flemish* or canad* or ottawa* or british columbia* or colombie britannique* or vancouver* or alberta* or edmonton* or calgar* or saskatchewan* or	6398698

	regina* or saskatoon* or manitoba* or winnipeg* or ontari* or toronto* or quebec* or montreal* or new brunswick* or nouveau brunswick* or fredericton* or nova scotia* or nouvelle ecosse* or halifax* or haligonian* or prince edward island* or ile du prince edouard* or pei or charlottetown* or newfoundland* or terre neuve* or labrador* or nfld or yukon* or whitehorse* or northwest territor* or territoires du nord ouest* or nwt or yellowknife* or nunavut* or iqaluit* or chile* or santiago or czech* or prague or denmark* or danish or dane* or faroe* or copenhagen or estonia* or tallinn or finland* or finnish* or helsinki* or france* or french* or paris* or marseille or lyon or lille or nice or toulouse or bordeaux or german* or deutschland* or berlin* or hamburg or munich or cologne or frankfurt or stuttgart or dusseldorf or greece* or hellenic* or greek* or athens or macedonia* or hungary* or hungarian* or budapest or iceland* or reykjavik or ireland* or irish* or dublin* or israel* or jerusalem or tel aviv or italy or italian* or rome or milan or naples or turin or sicily or japan* or tokyo or yokohama or osaka or nagoya or sapporo or kobe or kyoto or korea* or seoul or busan or daegu or daejeon or gwangju or incheon or ulsan or latvia* or riga or lithuania* or vilnius or luxembourg* or netherland* or holland* or dutch* or amsterdam or rotterdam or hague or new zealand* or aotearoa or wellington or auckland or maori or mexic* or norway* or norwegian* or oslo or poland* or polish or warsaw or krakow or wroclaw or lodz or portug* or lisbon or slovak* or bratislava or slovenia* or slovene* or ljubljana or spain* or spanish* or spaniard* or madrid or barcelona or catalonia* or valencia* or seville or zaragoza or malaga or basque or scandinavia* or sweden or swedish or swede* or stockholm or switzerland* or swiss* or zurich or geneva or bern or turkey or turkish or istanbul or constantinople or britain* or british* or united kingdom* or scotland* or scottish or wales* or welsh or england* or belfast or london or manchester or glasgow or birmingham or leeds or bradford or liverpool or alabama* or alaska* or arizona* or arkansas* or california* or colorado* or connecticut* or delaware* or florida* or georgia* or hawaii* or idaho* or illinois* or indiana* or iowa* or kansas* or kentucky* or louisiana* or maine* or maryland* or massachusetts* or michigan* or minnesota* or mississippi* or missouri* or montana* or nebraska* or nevada* or new hampshire* or new jersey* or new mexico* or new york* or north carolina* or north dakota* or ohio* or oklahoma* or oregon* or pennsylvania* or rhode island* or south carolina* or south dakota* or tennessee* or texas* or utah* or vermont* or virginia* or washington* or west virginia* or wisconsin* or wyoming* or montgomery* or juneau* or anchorage* or phoenix* or little rock* or sacramento* or los angeles* or san diego* or san francisco* or denver* or hartford* or dover* or tallahassee* or miami* or orlando* or atlanta* or honolulu* or boise* or springfield*	

	or chicago* or des moines* or topeka* or frankfort* or baton rouge* or new orleans* or augusta* or annapolis* or boston* or lansing* or detroit* or st?paul* or jackson* or jefferson city* or helena* or lincoln* or carson city* or reno* or las vegas* or concord* or trenton* or santa fe* or albany* or raleigh* or bismarck* or columbus* or oklahoma city* or salem* or harrisburg* or providence* or columbia* or peirre* or nashville* or austin* or dallas* or salt lake city* or montpelier* or richmond* or olympia* or seattle* or charleston* or madison* or cheyenne* or district of columbia* or usa or united states or europ*).tw,kw.	
27	25 and 26	476
28	limit 27 to ((english or french) and yr="2010 -Current")	349

Database(s): **Ovid MEDLINE(R) ALL** 1946 to April 08, 2021 (MCDMMedSmith2Rev3Final)
Search Strategy:

#	Searches	Results
1	*decision support techniques/	12314
2	(MCDA or MCDM or MADM or MAUM or MAVT or DCE or CRA or AHP).tw,kw,kf.	12114
3	((multicriteria or multi* criteria) adj3 (decision? or approach* or model* or technique*)).tw,kf.	1784
4	(multi* attribute adj3 (utility or value or decision)).tw,kw,kf.	382
5	(decision support adj4 (tool? or system?)).tw,kw,kf.	10092
6	(multiutility or multi utility or discrete choice experiment* or analytical hierarchy process* or comparative risk? or conjoint analy*).tw,kw,kf.	3997
7	or/1-6	37091
8	(priorit* or scor* or rank* or outrank* or order*).tw,kw,kf.	2470894
9	(weight* adj3 criteri*).tw,kw,kf.	1909
10	or/8-9	2472112
11	*public health/ or exp *population health/ or exp *environmental health/ or *one health/ or exp *veterinary medicine/ or exp *climate change/	118053
12	Communicable Diseases/pc or *Zoonoses/pc or Disease Outbreaks/pc	16164
13	exp *environmental impact assessment/ or exp *health impact assessment/	516
14	(communicable disease? or infectious disease? or infectious animal disease? or outbreak? or zoonos*).ti,kf. or (communicable disease? or infectious disease? or infectious animal disease? or outbreak? or zoonos*).ab. /freq=2	107666

15	((public or population or ecosystem? or environment* or animal? or ecolog*) adj2 health).tw,kw,kf.	349444
16	(one health or one medicine or veterinary medicine or climate change).tw,kw,kf.	53671
17	((environ* or health) adj2 assess*).tw,kw,kf.	54388
18	or/11-17	608450
19	and/7,10,18	753
20	Health Priorities/ or (health* adj2 priorit*).tw,kf.	19050
21	and/7,20	210
22	19 or 21	888
23	editorial/ or editorial.pt. or editorial?.tw,kw.	592702
24	22 not 23	886
25	exp australia/ or austria/ or exp baltic states/ or exp belgium/ or exp canada/ or chile/ or czech republic/ or exp "scandinavian and nordic countries"/ or exp france/ or exp germany/ or greece/ or hungary/ or exp ireland/ or israel/ or exp italy/ or exp japan/ or exp republic of korea/ or luxembourg/ or mexico/ or exp netherlands/ or exp new zealand/ or poland/ or exp portugal/ or slovakia/ or slovenia/ or exp spain/ or exp switzerland/ or turkey/ or exp united kingdom/ or exp united states/ or (australia* or new south wales or queensland or tasmania or victoria or sydney or melbourne or brisbane or adelaide or austria* or vienna or viennese* or belgium* or belgian* or brussels or flemish* or canad* or ottawa* or british columbia* or colombie britannique* or vancouver* or alberta* or edmonton* or calgar* or saskatchewan* or regina* or saskatoon* or manitoba* or winnipeg* or ontari* or toronto* or quebec* or montreal* or new brunswick* or nouveau brunswick* or fredericton* or nova scotia* or nouvelle ecosse* or halifax* or haligonian* or prince edward island* or ile du prince edouard* or pei or charlottetown* or newfoundland* or terre neuve* or labrador* or nfld or yukon* or whitehorse* or northwest territor* or territoires du nord ouest* or nwt or yellowknife* or nunavut* or iqaluit* or chile* or santiago or czech* or prague or denmark* or danish or dane* or faroe* or copenhagen or estonia* or tallinn or finland* or finnish* or helsinki* or france* or french* or paris* or marseille or lyon or lille or nice or toulouse or bordeaux or german* or deutschland* or berlin* or hamburg or munich or cologne or frankfurt or stuttgart or dusseldorf or greece* or hellenic* or greek* or athens or macedonia* or hungary* or hungarian* or budapest or iceland* or reykjavik or ireland* or irish* or dublin* or israel* or jerusalem or tel aviv or italy or italian* or rome or milan or naples or turin or sicily or japan* or tokyo or yokohama or osaka or nagoya or sapporo or kobe or	4842743

	kyoto or korea* or seoul or busan or daegu or daejeon or gwangju or incheon or ulsan or latvia* or riga or lithuania* or vilnius or luxembourg* or netherland* or holland* or dutch* or amsterdam or rotterdam or hague or new zealand* or aotearoa or wellington or auckland or maori or mexic* or norway* or norwegian* or oslo or poland* or polish or warsaw or krakow or wroclaw or lodz or portug* or lisbon or slovak* or bratislava or slovenia* or slovene* or ljubljana or spain* or spanish* or spaniard* or madrid or barcelona or catalonia* or valencia* or seville or zaragoza or malaga or basque or scandinavia* or sweden or swedish or swede* or stockholm or switzerland* or swiss* or zurich or geneva or bern or turkey or turkish or istanbul or constantinople or britain* or british* or united kingdom* or scotland* or scottish or wales* or welsh or england* or belfast or london or manchester or glasgow or birmingham or leeds or bradford or liverpool or alabama* or alaska* or arizona* or arkansas* or california* or colorado* or connecticut* or delaware* or florida* or georgia* or hawaii* or idaho* or illinois* or indiana* or iowa* or kansas* or kentucky* or louisiana* or maine* or maryland* or massachusetts* or michigan* or minnesota* or mississippi* or missouri* or montana* or nebraska* or nevada* or new hampshire* or new jersey* or new mexico* or new york* or north carolina* or north dakota* or ohio* or oklahoma* or oregon* or pennsylvania* or rhode island* or south carolina* or south dakota* or tennessee* or texas* or utah* or vermont* or virginia* or washington* or west virginia* or wisconsin* or wyoming* or montgomery* or juneau* or anchorage* or phoenix* or little rock* or sacramento* or los angeles* or san diego* or san francisco* or denver* or hartford* or dover* or tallahassee* or miami* or orlando* or atlanta* or honolulu* or boise* or springfield* or chicago* or des moines* or topeka* or frankfort* or baton rouge* or new orleans* or augusta* or annapolis* or boston* or lansing* or detroit* or st?paul* or jackson* or jefferson city* or helena* or lincoln* or carson city* or reno* or las vegas* or concord* or trenton* or santa fe* or albany* or raleigh* or bismarck* or columbus* or oklahoma city* or salem* or harrisburg* or providence* or columbia* or peirre* or nashville* or austin* or dallas* or salt lake city* or montpelier* or richmond* or olympia* or seattle* or charleston* or madison* or cheyenne* or district of columbia* or usa or united states or europ*).tw,kf.	
26	24 and 25	417
27	limit 26 to (yr="2010 -Current" and (english or french))	329

SCOPUS

((((TITLE-ABS-KEY ((multicriteria OR "multi* criteria") W/3 (decision? OR approach* OR model* OR technique*))) OR (TITLE-ABS-KEY ("multi* attribute" W/3 (utility OR value OR decision))) OR (TITLE-ABS-KEY ("decision support" W/4 (tool? OR system?))) OR (TITLE-ABS-KEY (multiutility OR "multi utility" OR "discrete choice experiment*" OR "analytical hierarchy process*" OR "comparative risk?" OR "conjoint analy*"))) AND ((TITLE (scor* OR rank* OR outrank* OR order*)) OR (TITLE-ABS (priorit*)) OR (TITLE (weight* W/3 criteri*))) AND ((TITLE ("communicable disease?" OR "infectious disease?" OR "infectious animal disease?" OR outbreak? OR zoonos* OR "climate change")) OR (TITLE-ABS-KEY ((public OR population OR ecosystem? OR environment* OR animal? OR ecolog*) W /2 health)) OR (TITLE-ABS-KEY ("one health" OR "one medicine" OR "veterinary medicine")) OR (TITLE-ABS-KEY ((environ* OR health) W/2 assess*)))) OR (((TITLE-ABS-KEY ((multicriteria OR "multi* criteria") W/3 (decision? OR approach* OR model* OR technique*))) OR (TITLE-ABS-KEY ("multi* attribute" W/3 (utility OR value OR decision))) OR (TITLE-ABS-KEY ("decision support" W/4 (tool? OR system?))) OR (TITLE-ABS-KEY (multiutility OR "multi utility" OR "discrete choice experiment*" OR "analytical hierarchy process*" OR "comparative risk?" OR "conjoint analy*"))) AND (TITLE-ABS-KEY (health* W/2 priorit*)))) AND ((TITLE-ABS-KEY (australia* OR austria* OR belgium OR belgian OR canada OR canadian OR chile OR czech OR denmark OR danish OR estonia OR finland OR finnish OR france OR french OR german* OR greece OR greek OR hungary OR hunarian OR iceland* OR ireland OR irish OR israel OR israeli OR italy OR italian OR japan* OR korea* OR latvia* OR luxembourg OR mexico OR mexican OR netherland OR dutch OR "new zealand" OR nz OR "new zealander" OR norway OR norwegian OR poland OR polish OR portugal OR portuguese OR slovakia OR slovak OR slovenia* OR spain OR spanish OR sweden OR swedish OR switzerland OR swiss OR turkey OR turkish OR britain OR british OR {united kingdom} OR uk OR scotland OR scottish OR wales OR welsh OR england OR {northern ireland} OR {united states} OR usa OR american OR america OR europ*))) AND PUBYEAR > 2009 AND (LIMIT-TO (LANGUAGE , "English")) 215 results

No French language articles found, no editorials found
